# Optical Fiber Sensor for Temperature and Strain Measurement Based on Multimode Interference and Square-Core Fiber

**DOI:** 10.3390/mi12101239

**Published:** 2021-10-13

**Authors:** Kun Wang, Xingchen Dong, Patrick Kienle, Maximilian Fink, Wolfgang Kurz, Michael H. Köhler, Martin Jakobi, Alexander W. Koch

**Affiliations:** Department of Electrical and Computer Engineering, Institute for Measurement Systems and Sensor Technology, Technical University of Munich, Theresienstraße 90, 80333 Munich, Germany; xingchen.dong@tum.de (X.D.); patrick.kienle@tum.de (P.K.); max.fink@tum.de (M.F.); wolfgang.kurz@tum.de (W.K.); michael.koehler@tum.de (M.H.K.); m.jakobi@tum.de (M.J.); a.w.koch@tum.de (A.W.K.)

**Keywords:** multimode interference, square-core fiber, temperature measurement, strain measurement, optical fiber sensors

## Abstract

A variety of specialty fibers such as no-core fiber (NCF) have already been studied to reveal their sensing abilities. In this work, we investigate a specialty fiber, square-core fiber, for temperature and strain sensing. A simple single-mode–multimode–single-mode (SMS) fiber sensor was fabricated, consisting of a 30-cm-long square-core fiber. The experimental results indicate that the maximal wavelength-temperature and wavelength-strain sensitivities are −15.3 pm/${}^{\circ }$C and −1.5 pm/με, respectively, while the maximal power-temperature and power-strain sensitivities are 0.0896 dBm/${}^{\circ }$C and 0.0756 dBm/με. Analysis of the results suggests that the fiber sensor has the potential to be used as a high-sensitivity temperature sensor with a low strain sensitivity.

## 1. Introduction

Optical fiber sensors have been a promising sensing device for decades due to their intrinsic characteristics, such as compact size, fast response, low cost, and insensitivity to electromagnetic fields [1,2,3,4]. A wide variety of configurations have been successfully reported according to the development of optical fiber fabrication techniques, for instance, based on fiber Bragg gratings (FBGs) [5], long-period gratings (LPGs) [6], Raman scattering [7], and Brillouin scattering [8]. The fabrication of these configurations usually requires sophisticated devices that result in relatively high costs. To address these factors, a simpler structure based on multimode interference was first reported by Mehta et al. in 2003 [9]. Since then, the multimode interference effect, which exists in multimode waveguides, has been comprehensively investigated. The basic optical fiber structure to fabricate a multimode interference device is a so-called single-mode–multimode–single-mode (SMS) fiber sensor, consisting of a short segment of multimode fiber (MMF) sandwiched between two single-mode fibers (SMFs).

Single-mode–multimode–single-mode (SMS) fiber structure has received significant attention in the last years because of its unique spectral features, high sensitivity, easy fabrication, and potential low costs [10,11]. It also shows great compatibility with other photonic devices and structures. For example, it can easily connect to other fiber structures with great flexibility to achieve better sensor performance such as higher sensitivity or multi-parameter measurement [12,13]. As such, it can perform flexibly as a variety of sensors in different scenarios, including temperature sensors [14], curvature sensor [15], refractive index sensors [16,17], humidity sensors [18], position sensors [19], strain sensors [20], and biomedical sensors [21].

Due to the high demand for sensing stability and measurement accuracy of fiber sensors, several effective post-processing technologies have been studied, such as tapering [22], etching [23], and polishing [24]. These approaches often require high-precision apparatus that drastically raise the manufacturing costs and difficulties, such as a high-accuracy fiber side-polishing system. An alternative approach, which can fulfill this demand with low costs and less complexity, is to consider specialty fibers by studying their feasibility and sensing performance. Some multimode-interference-based fiber sensors using specialty fibers have been reported to reveal the advantages of studying specialty fibers. Zhao et al. have fabricated a multimode-interference-based fiber sensor by splicing two segments of SMF and a 5-cm-long hollow core fiber (HCF) via two abrupt tapered joints. A temperature sensitivity of 0.0123 nm/${}^{\circ }$C and a curvature sensitivity of −5.05 dB/m${}^{-1}$ have been obtained [25]. Another multimode-interference-based fiber sensor consisting of cascaded MMF and photonic crystal fiber (PCF) fusion spliced with two sections of SMF was demonstrated by Yang et al., which has obtained a strain sensitivity of −14.89 pm/με and a temperature sensitivity of −243.8 pm/${}^{\circ }$C [26].

Here, we investigated the sensing performance of a simple multimode-interference-based fiber sensor containing a specialty fiber, the square-core fiber, for temperature and strain measurement. Square-core fiber produces an optical beam with uniform intensity over the core area because the shape of the core promotes mode mixing as light propagates through the fiber. It results in an even distribution of spatial modes in the output beam; therefore, it is ideal for applications such as laser machining. Further, square-core fiber also offers reduced focal ratio degradation, which means the square core shape is free from the input–output correlations, showing great potential for imaging and spectroscopy applications. These features of square-core fiber have been demonstrated by Velsink et al. by investigating the core shape, i.e., square core shape and circular core shape, for wavefront shaping application where a focus is formed at the output of the MMF [27]; however, the sensing ability of square-core fiber has not been experimentally studied. In this work, we demonstrated that the sensing performance of square-core fiber is promising. In addition, as the square core shape has not been experimentally proved, a comparison to other specialty fibers, which are mostly circular core shapes, is also presented. The experimental results also show that this fiber sensor can exhibit great potential applications in strain-insensitive temperature measurement.

## 2. Sensor Fabrication and Principle

A simple SMS fiber structure implementing the square-core fiber is proposed and studied. The general sensing principle of the SMS fiber sensor is based on the self-imaging effect in the multimode waveguide, i.e., multimode interference (MMI). The injected light is guided from the input SMF into the MMF and propagates along with the MMF core. The spot-size difference between the fundamental modes in the SMF and MMF excites a few lower modes in the MMF, which propagate with different propagation constants. The transmitted light in the MMF section generates multiple characteristic dips (or peaks) induced by multimode interference in its transmission spectrum [28]. At the second conjunction, these fields are coupled back to the fundamental mode of the output SMF. It needs to clarify that if the evanescent field of the SMS structure does not leak into the external media, it cannot sense the ambient changes. According to the detailed calculation [29,30], the sensing principle can be summarized as

(1) $$P_{out}={\left|\;A_{0}^{2}+A_{1}^{2}\;e^{i(\beta_{0}-\beta_{1})L}+A_{2}^{2}\;e^{i(\beta_{0}-\beta_{2})L}+\ldots \right|}^{2}\;,$$

where $P_{out}$ is the power in the output SMF, $A_{i}$ is the field amplitude of the *i*-th mode at the first SMF/MMF boundary, $\beta_{i}$ is the propagation constant of the *i*-th mode, and *L* is the length of the MMF. Equation (1) shows that the optical power in the output fiber is affected by physical changes caused by temperature and strain. A shift in the transmission spectrum occurs when temperature change and strain are applied to the MMF because they influence the inherent thermal expansion, photoelastic and thermo-optic effects of the fiber material, refractive indexes of the fiber, and fiber core diameter [31,32]; therefore, these changes can be quantitatively evaluated by measuring the shift in either power or spectral location of peaks (or dips).

**Figure 1:**
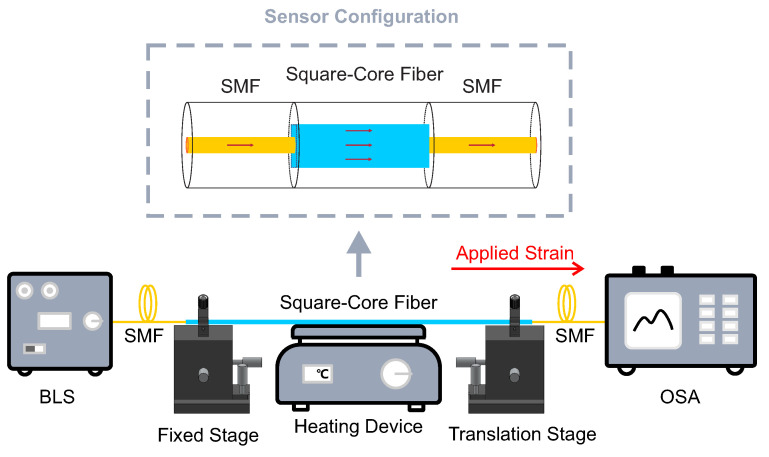
Schematic diagram of the experimental setup for temperature and strain measurement. The insert shows the schematic diagram of the fiber sensor; BLS, broadband light source; SMF, single-mode fiber; OSA, optical spectrum analyzer.

**Figure 2:**
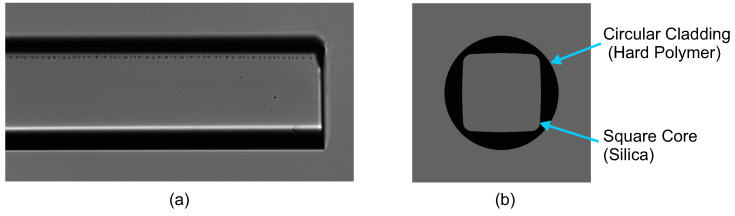
(**a**) Photo of square-core fiber from the top view. (**b**) Schematic diagram of the square core shape.

Figure 1 shows the schematic diagram of the experimental setup for temperature and strain measurement. The square-core fiber (FP150QMT, Thorlabs) features a 150 ± 10 μm × 150 ± 10 μm square silica core, surrounded by a ϕ225 μm circular polymer cladding. The square-core fiber with a length of 30 cm is used as the sensing region, as shown in Figure 2. As the cladding material comprises hard polymer, it is connected to two silica SMFs (size: 9/125 μm) by butt-coupling, i.e., both ends of the square-core fiber are connected to the ends of SMFs via FC/PC mating adaptors [33]. One end of the SMF connects to a broadband light source (BLS, central wavelength 1550 nm) emitting the incident light, while the end of the other SMF connects to an optical spectrum analyzer (OSA), which simultaneously detects the changes in the light spectrum. To investigate the response of the proposed fiber sensor to axial strain variation, the sample is placed and clamped on two translation stages, which can be accurately controlled and hence apply varying axial tensile stress on the sample according to a standard strain calculation equation [31]:

(2) $$\Delta \varepsilon =\frac{\Delta L}{L}\;,$$

where *L* is the initial distance between two translation stages and Δ*L* is the additional displacement when longitudinal stress is introduced to the proposed fiber sensor. The square-core fiber segment is also placed on a heating plate used as the heating device to control the temperature with 0.1 ${}^{\circ }$C resolution.

The transmission spectrum contains multiple dips caused by multimode interference within the spectrum range. In typical multimode interference structures, the interference fringes are being distorted due to multiple modes which are excited and participate in the interference. Although any of these dips (or peaks) can be used as a spectral indicator for sensing, the least distorted one is typically chosen to minimize the measurement error. In this work, we choose the one close to the central wavelength 1550 nm of the BLS. Note that the spectral distortion is significant in square-core fiber because of its imperfect end faces. In Figure 2a, it can be seen that the end face of square-core fiber is not perfect. This factor affects the coupling and the distortion in the transmission spectrum. Commonly, the specialty fibers can not be cleaved by standard cleaving devices, as the technology of cleaving specialty fibers has not been satisfactorily proved yet; however, if the imperfection is not in the fiber core region, the influence can be reduced or minimized. Further, using other methods, such as polishing, can improve the sensor performance. Despite these issues, this sensor structure is interesting because of its simple and inexpensive fabrication method and is therefore investigated in more detail.

## 3. Experiment and Discussion

**Figure 3:**
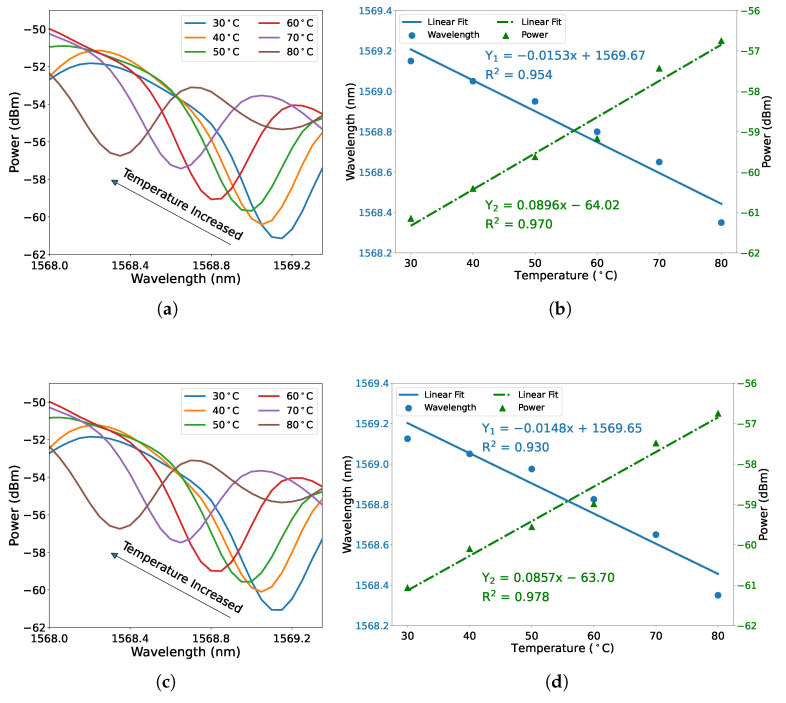
Measured dependence of spectral dip shifts on temperature in the range of 30 ${}^{\circ }$C to 80 ${}^{\circ }$C in steps of 10 ${}^{\circ }$C, (**a**) first round and (**c**) second round. Dip shift as a function of temperature, (**b**) first round and (**d**) second round. The blue solid line represents dip wavelength shift against temperature, and the green dash-dot line represents dip power against temperature.

The temperature dependence of the proposed fiber sensor was first measured without strain applied during the experiment. The square-core fiber section was heated in 10 ${}^{\circ }$C steps to stabilize the sensing ability from 30 ${}^{\circ }$C to 80 ${}^{\circ }$C. As shown in Figure 3a, the wavelength and power of the chosen dip change as the temperature changes. When the temperature increased, the dip shifted to shorter wavelengths accompanied by the transmission power increasing. The resulting changes in dip wavelength and transmission power versus temperature are plotted and fitted in Figure 3b. The fitting results of wavelength-temperature (blue solid line) and power-temperature (green dash-dot line) both exhibit a high linear regression coefficient value ($R^{2}$) when subject to linear regression analysis. It was determined that the wavelength-temperature sensitivity is −15.3 pm/${}^{\circ }$C with an $R^{2}$ value of 0.954, and the power-temperature sensitivity is 0.0896 dBm/${}^{\circ }$C with an $R^{2}$ value of 0.970. The experiment was carried out for the second round in the same temperature range, and the spectral dip shifts on temperature and the fitting results are as shown in Figure 3c,d, respectively. The measured wavelength-temperature sensitivity is −14.8 pm/${}^{\circ }$C with an $R^{2}$ value of 0.930, and the power-temperature sensitivity is 0.0857 dBm/${}^{\circ }$C with an $R^{2}$ value of 0.978. The absolute value of wavelength-temperature sensitivity is comparable with other specialty fiber sensors, such as the fiber sensor based on cascaded single-mode–no-core–single-mode (SNS) and single-mode–multimode–single-mode (SMS) fiber structure, the absolute value of obtained wavelength-temperature sensitivity is around 1.7 times than that [34].

**Figure 4:**
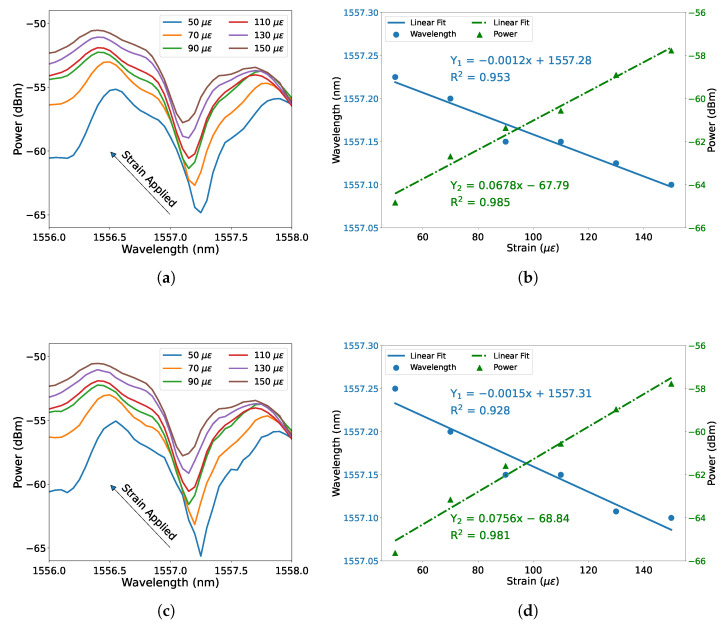
Measured dependence of spectral dip shifts on strain in the range of 50 με to 150 με in steps of 20 με, (**a**) first round and (**c**) second round. Dip shift as a function of strain, (**b**) first round and (**d**) second round. The blue solid line represents dip wavelength shift against strain, and the green dash-dot line represents dip power against strain.

Furthermore, the experiment of strain measurement was conducted at room temperature (averagely 20 ${}^{\circ }$C). The measured dependence of the spectral dip on strain is shown in Figure 4a. The spectral dip shifts to shorter wavelengths with increasing strain applied, whereas the transmission power increases. Figure 4b illustrates the dip wavelength and transmission power against strain, which also includes the linear fit of wavelength-strain (blue solid line) and power-strain (green dash-dot line), respectively. It can be seen that both fitting curves exhibit good linearity with high linear regression coefficient values ($R^{2}$). The wavelength-strain sensitivity is −1.2 pm/ε with an $R^{2}$ value of 0.953 and the power-strain sensitivity is 0.0678 dBm/ε with an $R^{2}$ value of 0.985. The experiment was carried out for the second round in the same strain range, and the spectral dip shifts on strain and the fitting results are as shown in Figure 4c,d, respectively. The measured wavelength-strain sensitivity is −1.5 pm/ε with an $R^{2}$ value of 0.928, and the power-strain sensitivity is 0.0756 dBm/ε with an $R^{2}$ value of 0.981. The absolute value of wavelength-strain sensitivity is comparable to the strain sensitivity of a thin core fiber implemented multimode interference fiber sensor with a core offset structure [35].

**Figure 5:**
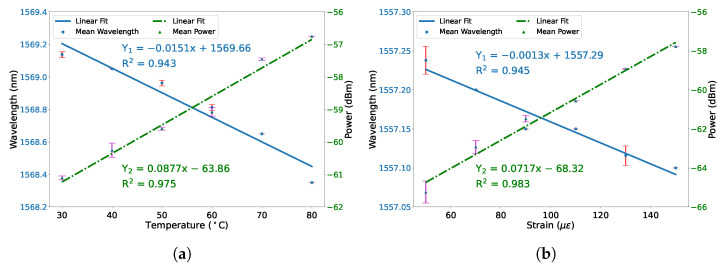
(**a**) Relation between average wavelength shifts and temperature, and the relation between average power changes and temperature. Error bar indicates the standard deviation. (**b**) Relation between average wavelength shifts and strain, and the relation between average power changes and strain. Error bar indicates the standard deviation.

The two-round reproducibility of the temperature and strain measurement is presented. Figure 5a shows the average wavelength-temperature sensitivity and power-temperature sensitivity are −15.1 pm/${}^{\circ }$C and 0.0877 dBm/${}^{\circ }$C, respectively. The maximal standard deviation of wavelength shifts is 0.0177 nm and of transmission power is 0.2174 dBm. Figure 5b shows the average wavelength-strain sensitivity is −1.3 pm/ε and average power-strain sensitivity is 0.0717 dBm/ε. The related maximal standard deviation of wavelength shifts and transmission power are 0.0176 nm and 0.5675 dBm, respectively.

Comparing Figure 3b and Figure 4b, the absolute value of wavelength-temperature sensitivity shows high-temperature measurability, while the small absolute value of wavelength-strain indicates that the proposed fiber sensor is less sensitive to applied strain. Therefore, the multimode interference in the square-core fiber can be potentially exploited to develop high-sensitivity temperature sensors with reduced strain sensitivity. Table 1 presents a comparison of the temperature measurability for different specialty fibers, which shows that the temperature sensing ability of square-core fiber is promising with a simple structure.

## 4. Conclusions

In conclusion, the sensing characteristics of square-core fiber were experimentally investigated in a simple SMS structure for temperature and strain measurement. The obtained maximal wavelength-temperature and wavelength-strain sensitivities are −15.3 pm/${}^{\circ }$C and −1.5 pm/με, respectively, with the maximal power sensitivities of 0.0896 dBm/${}^{\circ }$C and 0.0756 dBm/με for temperature and strain. The measurement results proved that square-core fiber is promising for sensing. Further, it also indicates that this sensor can exhibit great potential applications in strain-insensitive temperature measurement.

## Figures and Tables

**Table 1:** Comparison of temperature sensitivities with other multimode-interference-based optical fiber sensors using specialty fibers.

Optical Fiber Sensor Structure	Measurement Range	Sensitivity (max.)	Reference
SMF-HCF-SMF (with abrupt taper joints)	18–50 ${}^{\circ }$C	12.3 pm/${}^{\circ }$C	[25]
SMF-No-Core Fiber (NCF)- SMF-MMF-SMF	30–90 ${}^{\circ }$C	9.2 pm/${}^{\circ }$C	[34]
SMF-Tapered NCF-SMF	0–280 ${}^{\circ }$C	16.56 pm/${}^{\circ }$C	[36]
SMF-NCF-SMF (with FBG)	0–50 ${}^{\circ }$C	12.8 pm/${}^{\circ }$C	[37]
Tapered SMF-Micro MMF- Tapered SMF	35–60 ${}^{\circ }$C	0.028 dB/${}^{\circ }$C	[38]
SMF-Square-Core Fiber-SMF	30–80 ${}^{\circ }$C	−15.3 pm/${}^{\circ }$C 0.0896 dBm/${}^{\circ }$C	This work

## Data Availability

Not applicable.
